# US-Guided percutaneous core needle biopsy via the complete transhepatic approach: a reliable option for deep abdominal lesions

**DOI:** 10.1007/s00261-025-04958-0

**Published:** 2025-04-26

**Authors:** Halil Serdar Aslan, Kadir Han Alver

**Affiliations:** Pamukkale University, Denizli, Turkey

**Keywords:** Ultrasound-guided percutaneous core needle biopsy, Complete transhepatic approach, Challenging abdominal mass biopsies

## Abstract

**Purpose:**

To assess the feasibility, reliability, and diagnostic performance of ultrasound (US)-guided percutaneous core needle biopsy (PCNB) performed via the complete transhepatic approach (CTHa) for abdominal lesions.

**Materials and methods:**

This study included 71 patients (31 males, 40 females) with a mean age of 64.8 ± 13.9 years (range: 19–93) who underwent US-guided PCNB via the CTHa for abdominal lesions between January 2014 and December 2024. All biopsies were performed by interventional radiologists with at least five years of experience using a coaxial system and an 18-gauge automatic biopsy device. Patients were assessed for technical success, diagnostic yield, and complications, which were classified as major or minor based on the Society of Interventional Radiology (SIR) guidelines.

**Results:**

Tissue samples were successfully obtained in all cases, achieving a 100% technical success rate. Adequate material for pathological diagnosis was available in 63 of 71 patients (88.7%), while a definitive diagnosis could not be established in 11.3% of cases. Diagnostic yield was significantly influenced by lesion type (solid or mixed with cystic components) and anatomical location (*p* = 0.001 and *p* = 0.032, respectively). Complications occurred in 12.7% of patients, including 11.3% minor and 1.4% major complications. Univariate logistic regression analysis identified a history of malignancy, lesion size along the biopsy path, and the length of liver parenchyma traversed as significant predictors of complications (*p* = 0.012, 0.027 and 0.003 respectively). In the multivariate model, liver parenchyma length remained the only independent risk factor (*p* = 0.023).

**Conclusion:**

US-guided PCNB via the CTHa is a safe and effective option for abdominal lesions when extrahepatic access is not feasible. While longer liver tissue traversal increases the risk of minor complications, no major adverse events were observed. Careful procedural planning and consideration of lesion location and cystic content are essential to optimize diagnostic yield.

**Graphical Abstract:**

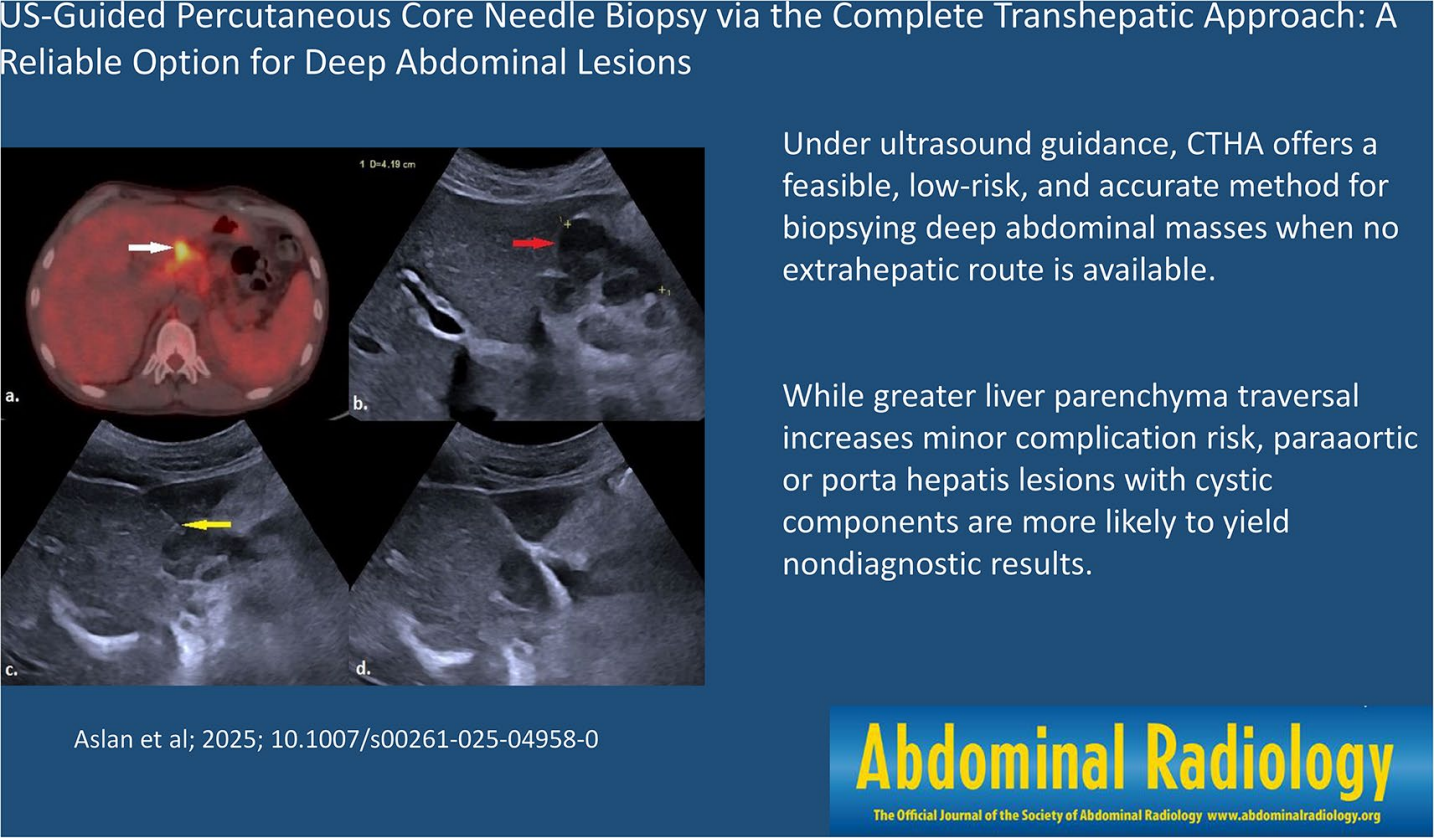

## Introduction

Despite remarkable progress in imaging technologies, biopsy remains the gold standard for the definitive diagnosis of abdominal lesions that cannot be conclusively characterized through radiological methods. Image guided percutaneous core needle biopsy (PCNB), a widely utilized and minimally invasive technique, has demonstrated its safety and efficacy, playing a pivotal role in establishing a diagnosis and guiding treatment planning [1, 2]. Although the majority of PCNBs for abdominal lesions can be performed smoothly using well-established and standardized techniques, some cases pose considerable technical challenges. These difficulties may arise from factors such as the lesion’s close proximity to surrounding vessels, the presence of overlying or intervening organs, poor lesion conspicuity due to minimal contrast with adjacent tissues, small lesion size, or respiratory motion causing displacement of lesions located near the diaphragm. A range of techniques and technologies, such as selecting different imaging modalities, adjusting the computed tomography (CT) gantry angle, administering intravenous contrast, optimizing patient positioning, employing hydrodissection, utilization of curved needles or pre-procedural image fusion, can individually or collectively address these challenges, enabling safe access to abdominal masses otherwise deemed unsuitable for PCNB. When no alternative routes are feasible, transgressing organs such as the liver, stomach, small bowel, kidney, lung, pleura, or even major blood vessels like the inferior vena cava (IVC) may be considered to facilitate access to challenging abdominal lesions [3–6].

The liver serves as an excellent acoustic window, allowing superior ultrasound (US) penetration and providing clear visualization of lesions located in the portal hilum, peripancreatic and paraduodenal regions, lesser sac, paraaortic and paracaval areas, right adrenal gland, and upper pole of right kidney. US-guided PCNB performed via a transhepatic approach offers several advantages, including real-time needle movement visualization, broad availability, cost-effectiveness, and significantly shorter procedure times. These benefits position US-guided PCNB as a compelling alternative to CT, fluoroscopy, and magnetic resonance imaging (MRI)-guided procedures, which are limited by longer procedure durations, higher costs, radiation exposure with CT and fluoroscopy, the need for MRI-compatible materials, and the inability to visualize needle movement in real time with CT and MRI. Moreover, US guidance, which allows multiple approach angles and flexible patient positioning, is crucial for planning the most reliable and shortest transhepatic access route by visualizing major vascular structures and the biliary system without the need for intravenous contrast.

Although the term ‘transhepatic’ is generally intended to describe procedures involving complete liver traversal, it is also ambiguously applied to procedures where only single capsule penetration occurs, such as liver parenchymal biopsies or percutaneous transhepatic cholangiography, potentially leading to conceptual confusion. By contrast, this study, along with similar investigations into definitive transhepatic approaches involving double capsule penetration, focuses on procedures requiring the traversal of the liver capsule twice. To address this ambiguity, we propose defining such procedures as a “complete transhepatic approach (CTHa)”.

Although the CTHa for PCNB is considered safe and effective, it remains insufficiently explored in the literature. While some studies have investigated CT-guided biopsies of abdominal masses, particularly right adrenal lesions, via the CTHa, these studies are generally dated and, in some cases, employed fine-needle aspiration biopsy techniques, which are now largely obsolete [7–9]. Furthermore, only a few studies with a limited number of cases have evaluated US-guided PCNB using CTHa, consistently confirming its feasibility and reliability [10, 11]. Therefore the aim of this study is to evaluate the feasibility, efficacy, and safety of US-guided PCNB of abdominal lesions performed via the CTHa, to identify potential factors and key determinants influencing diagnostic yield and procedural complications.

## Materials and methods

### Patient selection

This retrospective study received approval from our institutional review board and commenced following authorization from the local ethics committee (Approval date and number: 11/12/2024, E-60116787-020-622736). Due to its retrospective design, the requirement for informed consent was waived. However, at our institution, written informed consent was routinely obtained from all patients who agreed to undergo an abdominal mass biopsy after receiving comprehensive information. A total of 71 adult patients (aged 18 years or older) referred to the interventional radiology suite for abdominal mass biopsy between January 2014 and December 2024 were included in the study. Out of procedures performed using the CTHa route, biopsies performed under CT guidance (*n* = 17), cyst/collection/abscess drainage (*n* = 12), biopsies with fewer than three tissue samples obtained for any reason (*n* = 8), procedures without recorded images, inaccessible images, or those with poor/insufficient ultrasound image quality that precluded evaluation (*n* = 4), and fine-needle aspiration biopsy (FNAB) procedures (*n* = 3) were excluded. To maintain methodological consistency and ensure a homogeneous study population, only cases in which three core samples were obtained were included.

### Prebiopsy preparation

On the day of the biopsy, all patients underwent a complete blood count and coagulation panel. The pre-procedure requirements included a platelet count greater than 50,000/mL and an International Normalized Ratio (INR) below 1.5. In patients with cirrhosis or chronic liver disease, an extrahepatic route was used instead of the CTHa. Anticoagulant/antiplatelet therapy was reviewed, and adjustments, including cessation, bridging therapy, and reinitiation, were made in accordance with Society of Interventional Radiology (SIR) and Cardiovascular and Interventional Radiological Society of Europe (CIRSE) recommendations [1, 12, 13].

### Biopsy procedure

All biopsy procedures were performed under US (ACUSON Sequoia, Siemens; Aplio 500, Toshiba; LOGIQ E9, General Electric) guidance by two interventional radiologists with a minimum of 5 years of experience, following a consensus that the CTHa was the most optimal technique for accessing lesions. The CTHa was generally used only when accessing the lesion via an extrahepatic route was not feasible, such as in cases where intestinal or gastric gas, or bony structures, obstructed visibility or accessibility, or when the risks associated with the extrahepatic route (e.g., bowel injury risk, the presence of infected areas in the biopsy pathway) were considered higher than those of the transhepatic approach. Additionally, it was preferred when the lesion’s location necessitated traversing critical structures such as the colon or pancreas, making an extrahepatic approach impractical. In most cases, the right lobe of the liver was traversed to reach lesions in the right adrenal gland, right para-aortic/paracaval regions or the upper pole of the right kidney. Similarly, the left lobe was commonly used for lesions located in the pancreas. For lesions in the portal hilum/paraduedonal region, access through either the right or left lobe was chosen based on the specific location and patient anatomy. Patients were positioned in the supine or left lateral decubitus position, with the right arm placed behind the head. The procedure was performed under aseptic conditions, including skin disinfection with 10% povidone-iodine and the application of sterile drapes.

Local anesthesia (10–20 mL of 2% prilocaine hydrochloride) was administered subcutaneously, and around the superficial surface of the liver capsule—specifically the portion to be punctured during the initial capsule entry—using a 21-gauge needle. To ensure adequate anesthesia of the deeper portion of the liver capsule—particularly the area adjacent to the target lesion—an additional 10–20 mL of 2% prilocaine hydrochloride was administered after positioning the 17-gauge coaxial introducer just proximal to the second liver capsule penetration. At this point, the inner stylet was removed, and the anesthetic was delivered around the inner surface of the capsule near the lesion, helping to ensure sufficient analgesia and patient comfort during the procedure. Following this step, the inner stylet was reinserted, and the introducer advanced through the now-anesthetized deep capsule to access the lesion. In routine practice, biopsy procedures were performed under local anesthesia; however, for patients with severe anxiety or those deemed unable to tolerate the procedure with local anesthesia, the procedure was planned to be performed under conscious sedation (Fentanyl, 25–50 µg [maximum 100 µg]; Midazolam, 0.5–1 mg [maximum 5 mg]) in consultation with the anesthesia team [14, 15].

Tissue specimens were collected from the targeted lesion using an 18-gauge spring-loaded core biopsy device (Maxcore, Bard Biopsy Systems, Tempe, Arizona), which allowed for the acquisition of a 22 mm core sample. The coaxial technique was consistently utilized primarily to reduce the risk of bleeding complications by minimizing repeated penetration of the liver capsule, while also lowering the likelihood of needle tract tumor seeding. In all cases, autologous blood clot embolization of the tract was performed to mitigate the risk of bleeding complications. Major hepatic vessels, the liver hilum, bile ducts, gallbladder, and hepatic lesions such as cysts or hemangiomas were meticulously avoided during needle placement under the guidance of gray-scale or color Doppler US. After planning to reach the target lesion while minimizing the amount of liver tissue traversed and avoiding transgression of the described structures, a 17-gauge introducer was inserted into the target lesion using the CTHa. The inner stylet of the introducer was then removed, and three tissue samples were obtained from different regions of the lesion using an 18-gauge spring-loaded core biopsy device. In tumors with central necrosis, the peripheral areas containing soft tissue components of the lesion were targeted instead of the necrotic regions. Due to the unavailability of cytotechnologists during the procedure, the adequacy of the biopsy specimens was not routinely assessed on-site. The core biopsy samples were placed in a sterile container filled with 10% formalin and sent to the pathology department for histopathological examination, accompanied by a summary of the relevant clinical information.

### Postbiopsy monitorization and complications

Patients were monitored in the recovery room for at least six hours after the procedure. Depending on the biopsy site, they were positioned in either a supine or right lateral decubitus position. Vital signs (pulse rates, pulse oximetry values and blood pressures) were checked at 30-minute intervals for the first two hours and then hourly for the remaining time, while the puncture site was inspected for bleeding. During the observation period, all patients showing signs of hypotension or tachycardia, or experiencing symptoms such as moderate to severe pain, vomiting, or shortness of breath, underwent an urgent bedside ultrasound examination. If deemed necessary by the radiologist, a CT scan was subsequently performed for further evaluation. At the end of the observation period, radiologists performed an abdominal ultrasound on all patients, who were discharged if they were hemodynamically stable, exhibited no signs of bleeding, and reported no severe symptoms. Prior to discharge, each patient received explicit guidance to promptly reach out to the hospital if they experienced symptoms like chills, elevated temperature, respiratory distress, pain in the abdomen, shoulder, or chest, hemorrhage, swelling at the biopsy location, or the presence of blood in their stool. Complications were assessed based on records from the initial 6-hour observation period in our clinic, as well as medical charts covering the first month after discharge to account for delayed bleeding or late-onset complications. Post-biopsy complications were classified as minor or major in accordance with the SIR guidelines. Minor complications included those that required no therapy or nominal therapy without significant consequences, such as overnight admission for observation. Major complications were defined as those necessitating therapy or hospitalization, ranging from minor hospitalization lasting less than 48 h to major interventions, prolonged hospitalization exceeding 48 h, permanent adverse outcomes, or death [16].

### Radiologic evaluation and analysis

**Fig. 1:**
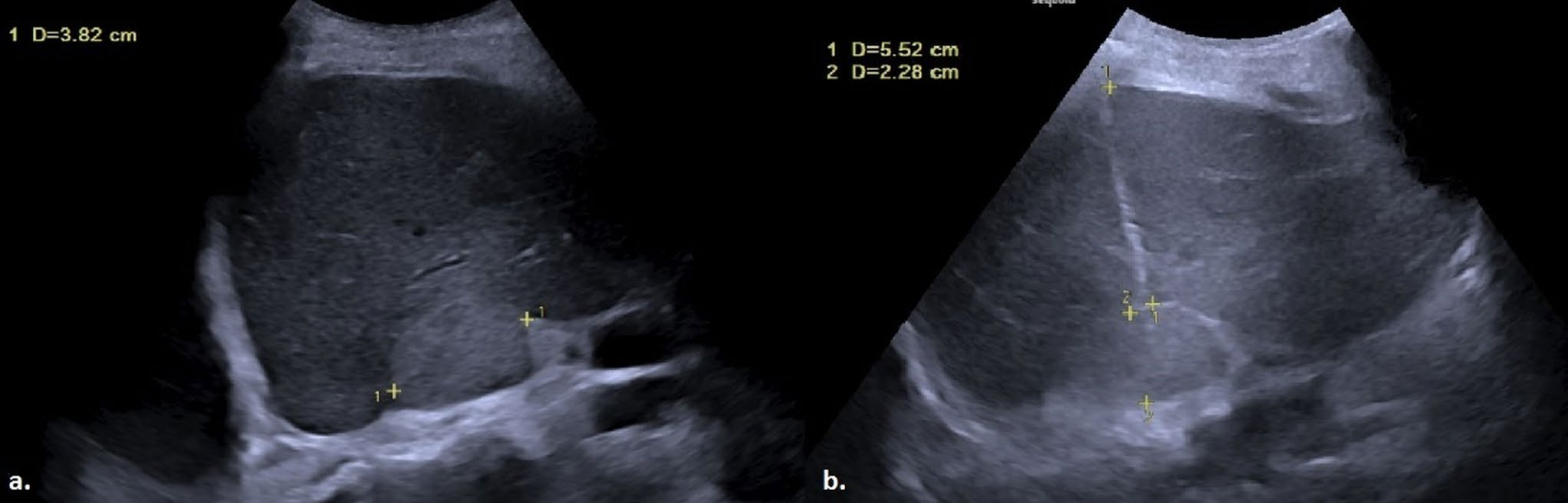
**a**) A mildly heterogeneous hyperechoic mass lesion, measuring a maximum diameter of 3.82 cm, is observed in the right adrenal gland. **b**) During the biopsy of the lesion performed via the CTHa, the distance traversed by the 17-gauge introducer through the right lobe of the liver parenchyma and the maximum diameter of the lesion along the biopsy trajectory were calculated as 5.52 cm and 2.28 cm, respectively (CTHa: Complete Transhepatic Approach)

Patient demographic data, clinical history, and biopsy pathology results were retrieved from electronic medical records. Follow-up information, including the maximum duration until the most recent imaging examination involving the biopsy site and any evidence of tumor seeding along the biopsy tract, was also reviewed. Additionally, data on patients who passed away during this period were extracted from the system. Radiological data were recorded for each procedure, including the location of the lesion, classified as right adrenal gland, upper pole of the right kidney, right para-aortic/paracaval region, portal hilum, pancreatic head, or pancreatic neck and body. The maximum size of the lesions, their dimensions along the biopsy trajectory (defined as the distance along the needle path in the firing direction of the biopsy device), the liver lobe traversed during the procedure (right or left), the length of liver parenchyma crossed by the needle and the nature of the lesions (entirely solid or containing cystic areas) were documented (Fig. 1). These measurements were retrospectively obtained by analyzing US images routinely recorded and saved to the picture archiving and communication system (PACS) as part of standard practice during each procedure. US-guided biopsy imaging included pre-procedural scans to locate the lesion, real-time images acquired during the biopsy to ensure accurate targeting, and post-procedural scans to assess for any immediate complications.

Technical success was defined as the accurate placement of the biopsy needle within the target lesion and completion of the planned biopsy with a possible pathological evaluation. During the follow-up period, histopathological diagnoses of all biopsied lesions and information regarding whether tissue samples were considered diagnostic or non-diagnostic were also recorded.

### Statistical analysis

All statistical analyses were performed using SPSS version 25.0 (IBM SPSS Statistics for Windows, IBM Corp., Armonk, NY, USA). The normality of data distribution was assessed using skewness, kurtosis, the Kolmogorov–Smirnov test, and the Shapiro–Wilk test. Descriptive statistics were presented as frequency and percentage for categorical variables and as mean, standard deviation, minimum, and maximum for continuous variables.

The diagnostic yield of the procedure was evaluated based on pathology results. Associations between diagnostic yield, the presence of complications and variables such as age, gender, history of malignancy, target lesion location, imaging characteristics (lesion size, maximum dimension along the biopsy pathway, and lesion composition [entirely solid or containing cystic areas]), the liver lobe traversed by the needle (right or left), and the length of liver parenchyma crossed by the needle were analyzed using chi-square tests for categorical variables and either the Student’s t-test or the Mann-Whitney U test, depending on the normality of data distribution. Logistic regression analysis was conducted to determine the factors affecting the likelihood of complications. Each variable’s effect was individually evaluated during the univariate analysis. The multivariate logistic regression model was constructed to identify independent predictors of complications, including all relevant variables to control for potential confounding effects. Results were expressed as odds ratios (ORs) with corresponding 95% confidence intervals (CIs). A p-value < 0.05 was considered statistically significant for all tests.

## Results

This study included 71 patients (31 males, 40 females) with a mean age of 64.8 ± 13.9 years; (range: 19–93) who underwent US-guided abdominal mass biopsy through CTHa. The most frequently observed location was the porta hepatis (25/71; 35.2%), followed by the right adrenal gland (17/71; 23.9%) and the pancreatic neck and body (9/71; 12.7%). Other sites included the upper pole of the right kidney (6/71; 8.5%), the pancreatic head (6/71; 8.5%), and the lesser omentum and right paraaortic/paracaval region, each accounting for 4 out of 71 lesions (5.6%), in decreasing frequency. Of the 71 lesions, 49 (69%) were classified as solid, whereas 22 (31%) exhibited a mixed composition with one or more cystic components. Histopathological analysis revealed that pancreatic ductal adenocarcinoma was the most prevalent diagnosis (10/71 lesions, 14.1%), followed by metastatic lung adenocarcinoma (7/71 lesions, 9.9%) and Hodgkin lymphoma (6/71 lesions, 8.5%). Malignant lesions comprised the majority (53/71; 74.6%), while benign tumors accounted for 10/71 cases (14.1%). In 8/71 cases (11.3%), the pathological assessment was nondiagnostic. For a detailed breakdown of the histopathological results, type and locations of the biopsied lesions, please refer to Table 1.

**Table 1:** Locations, types and histopathological results of biopsied lesions

			Number (*n*)	Percentage (%)
Location		Porta hepatis	25	35,2%
		Right adrenal gland	17	23,9%
		Pancreatic neck and body	9	12,7%
		Upper pole of right kidney	6	8,5%
		Pancreatic head	6	8,5%
		Lesser omentum	4	5,6%
		Right paraaortic/paracaval region	4	5,6%
Lesion Nature		Solid	49	69,0%
		Mixed solid and cystic	22	31,0%
Pathology Results	Malign (*n* = 53; 74.6%)	Pancreatic ductal adenocarcinoma	10	14,1%
		Lung adenocarcinoma metastasis	7	9,9%
		Hodgkin lymphoma	6	8,5%
		Renal cell carcinoma	4	5,6%
		Lung squamous cell carcinoma metastasis	3	4,2%
		Diffuse large B-cell lymphoma	3	4,2%
		Lung small cell carcinoma metastasis	2	2,8%
		Hepatocellular carcinoma metastasis	2	2,8%
		Colon carcinoma metastasis	2	2,8%
		Mantle cell lymphoma	2	2,8%
		Breast cancer metastasis	2	2,8%
		Renal cell carcinoma metastasis	2	2,8%
		Gastric adenocarcinoma metastasis	1	1,4%
		Gastric adenocarcinoma	1	1,4%
		Gastrointestinal stromal tumor	1	1,4%
		Small intestine lymphoma	1	1,4%
		Dedifferentiated liposarcoma	1	1,4%
		Adrenal lymphoma	1	1,4%
		Adrenocortical carcinoma	1	1,4%
		Papillary renal cell carcinoma	1	1,4%
	Benign (*n* = 10; 14.1%)	Adrenal adenoma	2	2,8%
		Reactive lymph node	2	2,8%
		Granulomatous lymphadenitis	2	2,8%
		Retroperitoneal fibrosis	1	1,4%
		Tuberculous lymphadenopathy	1	1,4%
		Serous cystadenoma of pancreas	2	2,8%
	Nondiagnostic (*n* = 8; 11.3%)	Nondiagnostic	8	11,3%

Tissue samples were successfully collected for pathological diagnosis in all cases, resulting in a 100% technical success rate. Sufficient material for pathological assessment was available in 63 of 71 patients (88.7%). In 8 of 71 patients (11.3%), a definitive pathological diagnosis could not be made. Of these, three had mesenchymal tissue samples with fat and muscle, indicating normal histology, three exhibited features suspicious for malignancy, and two had specimens that were considered nondiagnostic upon pathological assessment. Biopsy was performed in 29 out of 71 patients (40.9%) with a prior history of malignancy. Among these, 20 cases had pathological findings consistent with their known primary cancer, while three were diagnosed with benign conditions (one adrenal adenoma and two reactive lymph nodes), and two were found to have an additional malignancy. In four patients with a known malignancy history, tissue samples were insufficient for a conclusive diagnosis. In 42 patients without a known history of malignancy, malignancy was identified in 31 cases. Meanwhile, seven patients were diagnosed with benign pathologies, including granulomatous lymphadenitis (*n* = 2), serous cystadenoma of the pancreas (*n* = 2), tuberculosis lymphadenitis (*n* = 1), adrenal adenoma (*n* = 1), and retroperitoneal fibrosis (*n* = 1). In four cases, the biopsy results were nondiagnostic.

Analyzing patient and lesion characteristics in relation to positive biopsy outcomes revealed that lesion type (solid or mixed with cystic components) and anatomical location significantly influenced diagnostic yield (*p* = 0.001 and *p* = 0.032, respectively). In contrast, factors such as patient gender, age, maximum lesion diameter, and history of malignancy showed no statistically significant association with diagnostic success (*p* > 0.05). Of the eight nondiagnostic results, six lesions were located in the porta hepatis and two in the right paraaortic/paracaval region, with seven exhibiting a mixed composition with cystic components and only one classified as entirely solid (Table 2).

**Table 2:** Comparison of demographics, clinical variables, lesion characteristics, and procedural details between patients with diagnostic and non-diagnostic histopathological results

	Pathology Results		*p*	
	Nondiagnostic (*n* = 8) (11.3%)	Diagnostic (*n* = 63) (88.7%)		
	*n* (%)	*n* (%)		
Gender				
Male	4 (50%)	27 (42.9%)	0.722	*
Female	4 (50%)	36 (57.1%)		
Age	65.3 ± 10.9	64.7 ± 14.4	0.928	**
Maximum diameter of lesion	43.6 ± 27.1	49.8 ± 25.2	0.506	**
Maximum diameter along the biopsy path	22.0 ± 4.9	34.6 ± 18.2	0.081	**
The length of liver parenchyma crossed	56.4 ± 24.8	58.6 ± 23.7	0.913	***
The liver lobe traversed				
Right	3 (37.5%)	31 (49.2%)	0.712	*
Left	5 (62.5%)	32 (50.8%)		
Lesion location				
Porta hepatis	6 (75%)	19 (30.2%)	**0.032**	*
Right adrenal gland	0	17 (27%)		
Pancreatic neck and body	0	9 (14.3%)		
Upper pole of right kidney	0	6 (9.5%)		
Pancreatic head	0	6 (9.5%)		
Lesser omentum	0	4 (6.3%)		
Right paraaortic/paracaval region	2 (25%)	2 (3.2%)		
Lesion type				
Solid	1 (12.5%)	48 (76.2%)	**0.001**	*
Mixed solid and cystic	7 (87.5%)	15 (23.8%)		
History of malignancy				
Yes	4 (50%)	25 (39.7%)	0.708	*
No	4 (50%)	38 (60.3%)		

*: Chi-square analysis; **: Mann Whitney U test; ***: Student t test

**Fig. 2:**
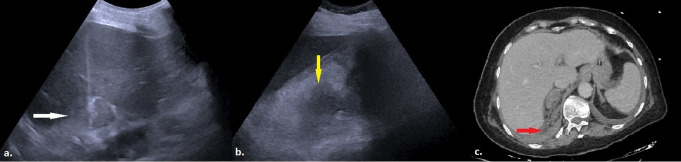
**a**) The image shows an ultrasound view obtained immediately after a biopsy performed via CTHa of a mass in the right adrenal region (white arrow). **b**) A control US examination performed during the observation period, revealed a heterogeneous hypoechoic area in the right retroperitoneum (yellow arrow), corresponding to the biopsy site, with an indistinct border from the lesion. **c**) An abdominal CT scan demonstrated hemorrhage in the retroperitoneal area surrounding the biopsied mass (red arrow). Pathological examination of the lesion confirmed metastasis of lung adenocarcinoma (CTHa: Complete Transhepatic Approach, US: Ultrasound; CT: Computed Tomography)

**Fig. 3:**
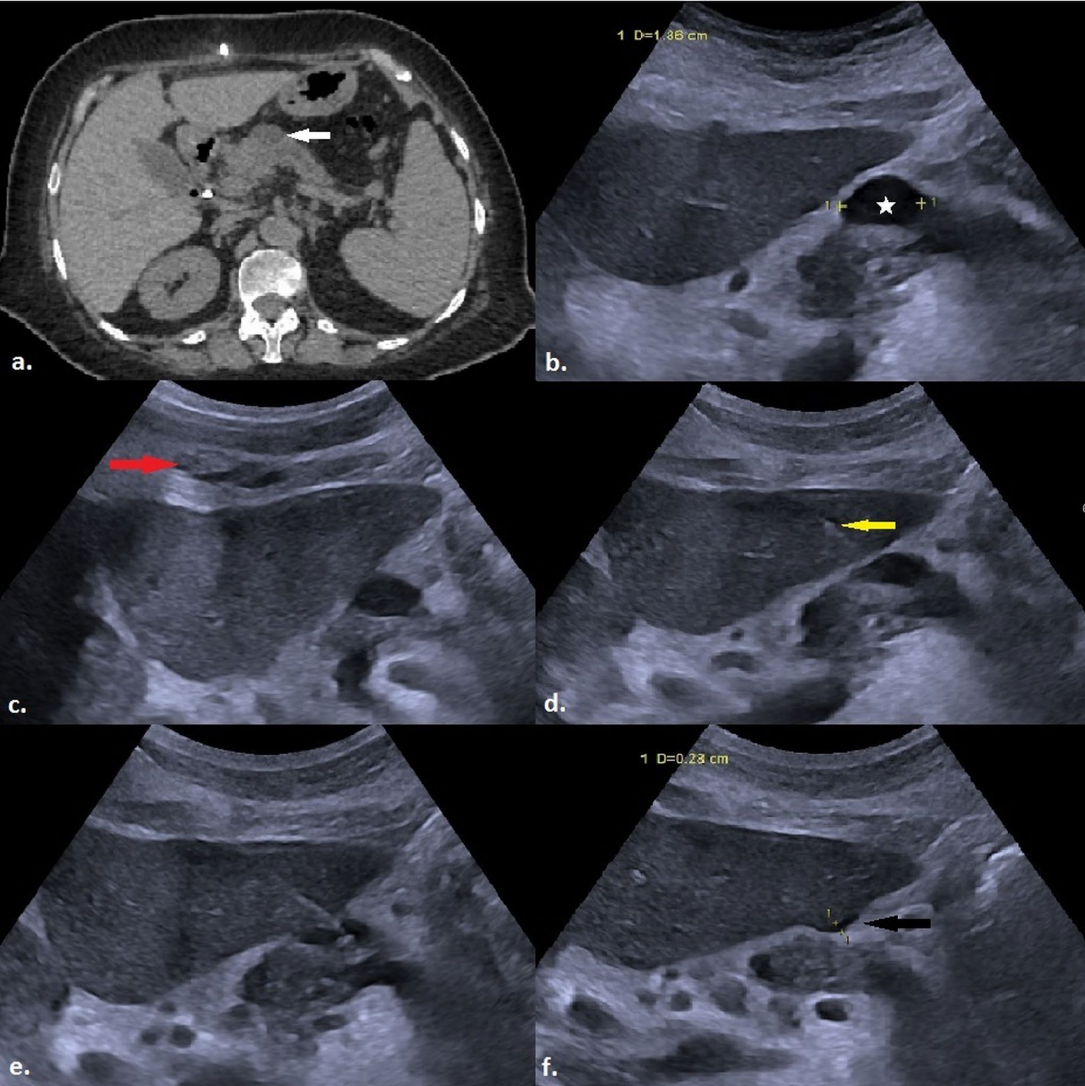
**a**) A well-defined hypodense lesion (white arrow) is observed in the axial section of a non-contrast abdominal CT scan, located at the neck-body junction of the pancreas. **b**) The maximum diameter of the same lesion (white star) is measured as 1.86 cm on US imaging. **c**) Prior to the biopsy planned under US guidance using the CTHa, local anesthesia is administered with approximately 10–20 ml of 2% prilocaine hydrochloride around the anterior liver capsule and subcutaneous tissue (red arrow). **d**) The 17-gauge introducer (yellow arrow) is advanced completely through the liver parenchyma to reach the lesion. **e**) US image recorded immediately after the biopsy procedure. **f**) Post-procedural control US examination reveals a minimal hemorrhage, measuring 0.23 cm in thickness, in the perihepatic area at the procedure site (black arrow). The patient, who showed no abnormalities during follow-up, was discharged without major complications. The pathological examination of the lesion was reported as granulomatous lymphadenitis (CTHa: Complete Transhepatic Approach, US: Ultrasound; CT: Computed Tomography)

**Fig. 4:**
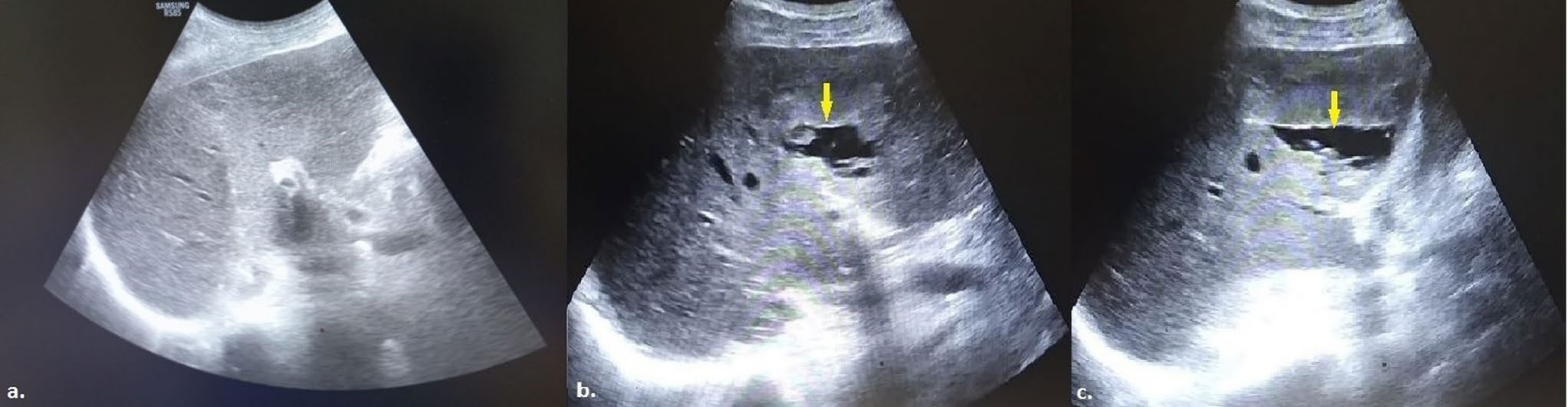
**a**) The image shows the biopsy procedure performed via CTHa on a nodular lesion (white arrow) detected in the right adrenal region during staging imaging of a patient diagnosed with gastric adenocarcinoma. **b**, **c**) Post-procedural control ultrasound (US) images demonstrate a localized intrahepatic hemorrhage (yellow arrows) within the liver. Pathological examination of the lesion revealed an adenoma (CTHa: Complete Transhepatic Approach, US: Ultrasound)

Complications were observed in 9 out of 71 patients (12.7%) during the recovery period, comprising eight minor complications (11.3%) and one major complication (1.4%). The major complication was a retroperitoneal hemorrhage that required no additional intervention other than the transfusion of two units of erythrocyte suspension (Fig. 2). Minor complications included four instances of mild perihepatic bleeding (5.6%) (Fig. 3), one case of intrahepatic hemorrhage (1.4%) (Fig. 4), a skin hematoma (1.4%), one episode of moderate pain (1.4%) that resolved without analgesic administration, and one occurrence of vasovagal reflex (1.4%). All minor perihepatic and intrahepatic hemorrhages were detected through follow-up ultrasound examinations performed at the end of the observation period. Examining patient and lesion characteristics in relation to the likelihood of complications indicated that a history of malignancy, the maximum lesion diameter along the biopsy trajectory, and the extent of liver parenchyma traversed were significant factors affecting complication risk (*p* = 0.003, 0.007, and < 0.0001, respectively) (Table 3). In the univariate binary logistic regression analysis, a history of malignancy, the maximum diameter of the lesion along the biopsy path, and the length of liver parenchyma traversed were found to significantly influence the likelihood of complications, with p-values ranging from 0.003 to 0.027. In the multivariate reduced model, the length of liver parenchyma crossed remained a significant independent factor in distinguishing between the two groups, with a p-value of 0.023 (Table 4).

**Table 3:** Comparison of demographics, clinical variables, lesion characteristics, and procedural details between procedures with and without complications

	Presence of Complication		*p*	
	Minor and Major Complication (*n* = 9) (12.7%)	No Complication (*n* = 62) (87.3%)		
	*n* (%)	*n* (%)		
Gender				
Male	4 (44.4%)	27 (43.5%)	0,960	*
Female	5 (55.6%)	35 (56.5%)		
Age	68.8 ± 10.4	64.2 ± 14.4	0,500	**
Maximum diameter of lesion	39.6 ± 13.8	50.5 ± 26.3	0,257	**
Maximum diameter along the biopsy path	19.8 ± 4.7	35.1 ± 18.0	**0**,**007**	**
The length of liver parenchyma crossed	86.7 ± 11.9	54.3 ± 22.1	**< 0.001**	***
The liver lobe traversed				
Right	7 (77.8%)	27 (43.5%)	0,077	*
Left	2 (22.2%)	35 (56.5%)		
Lesion location				
Porta hepatis	4 (44.4%)	21 (33.9%)	0,368	*
Right adrenal gland	5 (55.6%)	12 (19.4%)		
Pancreatic neck and body	0	9 (14.5%)		
Upper pole of right kidney	0	6 (9.7%)		
Pancreatic head	0	6 (9.7%)		
Lesser omentum	0	4 (6.5%)		
Right paraaortic/paracaval region	0	4 (6.5%)		
Lesion type				
Solid	6 (66.7%)	43 (69.4%)	0,871	*
Mixed solid and cystic	3 (33.3%)	19 (30.6%)		
History of malignancy				
Yes	8 (88.9%)	21 (33.9%)	**0**,**003**	*
No	1 (11.1%)	41 (66.1%)		

*: Chi-square analysis; **: Mann Whitney U test; ***: Student t test

**Table 4:** Logistic regression analysis of factors influencing the likelihood of complications

Dependent Variable: Presence of Complication	Univariate				Multivariate			
	*p*	OR	95% CI for OR		*p*	OR	95% CI for OR	
			Lower	Upper			Lower	Upper
Gender	0,960	1.037	0,254	4.236				
Age	0,360	1.028	0,969	1.089				
Maximum diameter of lesion	0,231	0,977	0,941	1.015				
Maximum diameter along the biopsy path	**0**,**027**	0,886	0,795	0,986	0,079	0,895	0,791	1.013
The length of liver parenchyma crossed	**0**,**003**	1.106	1.035	1.182	**0**,**023**	1.073	1.010	1.141
The liver lobe traversed	0,072	4.537	0,872	23.617				
Lesion type	0,871	1.132	0,256	5.008				
History of malignancy	**0**,**012**	15.619	1.830	133.333	0,091	7.841	0,718	85.657

A review of medical records revealed that 39 out of 71 patients who underwent biopsy had died an average of 303 ± 389 days (range; 10–1946 days) after the procedure. Additionally, during a follow-up period of 346 ± 462 days (range; 10–2340 days), no tumor seeding was detected along the biopsy tract.

## Discussion

**Fig. 5:**
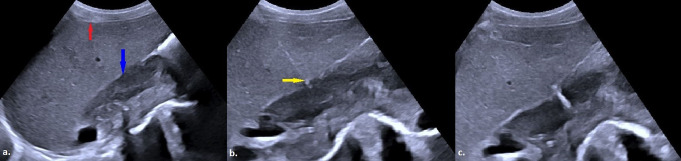
**a**) Ultrasound image shows a hypoechoic lesion (blue arrow) located in the right retroperitoneum. Local anesthesia was applied with 10–20 ml of 2% prilocaine hydrochloride (red arrow) to the subcutaneous tissue and the area adjacent to the anterior liver capsule before the biopsy. **b**) The lesion is accessed via CTHa using a 17-gauge introducer (yellow arrow). **c**) An ultrasound image recorded immediately after the biopsy procedure. Pathological examination of the lesion confirmed diffuse large B-cell lymphoma (CTHa: Complete Transhepatic Approach)

This study demonstrates that US-guided PCNB via the CTHa is a highly effective and safe technique when performed by experienced interventional radiologists, achieving a 100% technical success rate and an 88.7% diagnostic accuracy (Fig. 5). Our findings indicate that complications occurred in 12.7% of cases, with only 1.4% classified as major, highlighting the procedure’s favorable risk profile. Notably, lesions located in the porta hepatis and right paraaortic/paracaval regions, as well as those containing cystic components, were more likely to result in nondiagnostic pathology. Moreover, while primary malignancy, smaller lesion size along the biopsy trajectory, and increased liver parenchyma length traversed were associated with higher complication rates, multivariate analysis identified liver tissue length as the most significant independent predictor of complications.

**Fig. 6:**
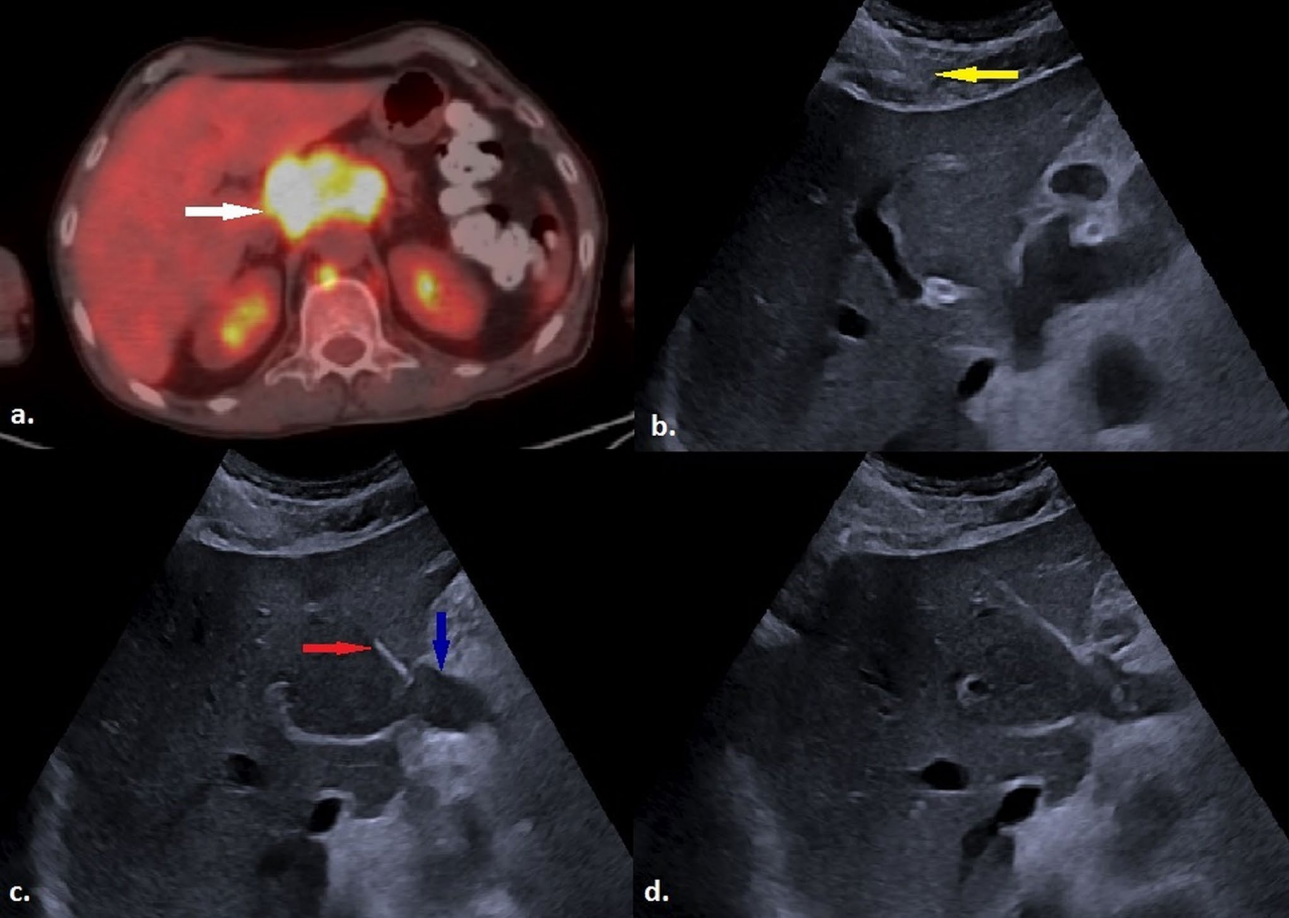
**a**) An axial PET/CT image shows conglomerate lymphadenopathy with high SUV uptake in the porta hepatis region (white arrow). **b**) Prior to the biopsy, local anesthesia is administered with 2% prilocaine hydrochloride using a 21-gauge needle to the subcutaneous tissue and around the anterior liver capsule (yellow arrow). **c**) A hypoechoic lymph node in the portal hilum (blue arrow) is accessed via CTHa using a 17-gauge introducer (red arrow). **d**) Ultrasound image obtained immediately after the biopsy procedure. Pathological examination of the lesion confirmed Hodgkin lymphoma. (PET/CT: Positron Emission Tomography / Computed Tomography; CTHa: Complete Transhepatic Approach; SUV: Standardized Uptake Value)

Although the literature suggests that transhepatic biopsy of abdominal lesions is a safe and effective approach, studies specifically investigating this technique remain scarce, with most having limited patient populations. For instance, Park et al. evaluated only 10 transhepatic biopsies, while Price et al. included just eight cases, and Onishi et al. analyzed 26 patients [7, 10, 11]. The small sample sizes in these studies limit the generalizability of their findings. Additionally, some studies employed techniques that differ significantly from contemporary biopsy methods, thereby restricting their applicability to current clinical practice. Onishi et al. utilized a CT-angiography system, exposing patients to ionizing radiation, whereas both Price et al. and Kocijancic et al. performed fine-needle aspiration biopsy (FNAB), which is now rarely used for intra-abdominal lesion biopsies [17]. Furthermore, the duration of post-biopsy monitoring was not explicitly reported in most studies, with Price et al. observing patients for only one hour, raising concerns about the potential for underreporting of complications, including delayed hemorrhage. While long-term follow-up data on tumor seeding are available in some studies, none specifically assessed late-onset complications beyond tumor implantation. Despite these methodological differences, our findings align with previously reported complication rates and diagnostic performance values (Fig. 6).

**Fig. 7:**
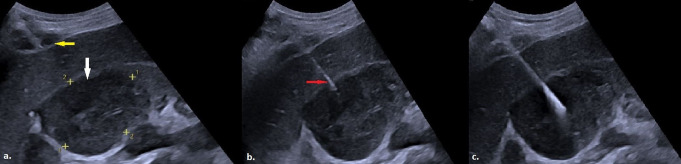
**a**) Ultrasound image demonstrates a mildly heterogenous hypoechoic mass lesion (white arrow) located at the upper pole of the right kidney. Before the biopsy, local anesthesia is applied using a 21-gauge needle to the subcutaneous tissue and the region surrounding the anterior liver capsule with 2% prilocaine hydrochloride (yellow arrow). **b**) The mass is accessed via CTHa using a 17-gauge introducer (red arrow). **c**) A US image obtained immediately following the biopsy procedure. Histopathological analysis confirmed the lesion as renal cell carcinoma (CTHa: Complete Transhepatic Approach; US: Ultrasound)

Ultrasound guidance should be the preferred modality for transhepatic abdominal mass biopsies due to its significant advantages. Real-time needle visualization allows precise differentiation of vascular structures, dilated bile ducts, and hepatic lesions, facilitating the avoidance of these critical structures without the need for contrast agents (Fig. 7). Most importantly, US-guided procedures eliminate patient exposure to ionizing radiation. Although various optimization strategies have been proposed to reduce radiation dose in CT-guided interventional procedures—including decreasing cranio-caudal scan length, reducing beam energy, lowering photon fluence, and increasing pitch—there is limited information on the extent to which these techniques are consistently applied in clinical practice [18–20]. Regardless of the optimization strategies used, patients inevitably receive a certain level of radiation exposure during CT-guided procedures. Studies have reported that the mean dose-length product (DLP) in CT-guided abdominal mass biopsies (including liver, adrenal, kidney, pancreas, and retroperitoneal/mesenteric mass biopsies) ranges from 9.8 to 19.7 mGy [21–23], which is comparable to the dose reported for diagnostic abdominal-pelvic CT scans (8–13 mGy) according to the American College of Radiology dose index registry and other recent studies [24, 25]. Given these considerations, our institution prioritizes ultrasound guidance for all feasible biopsies and reserves CT guidance only for cases where ultrasound guidance is not viable.

**Fig. 8:**
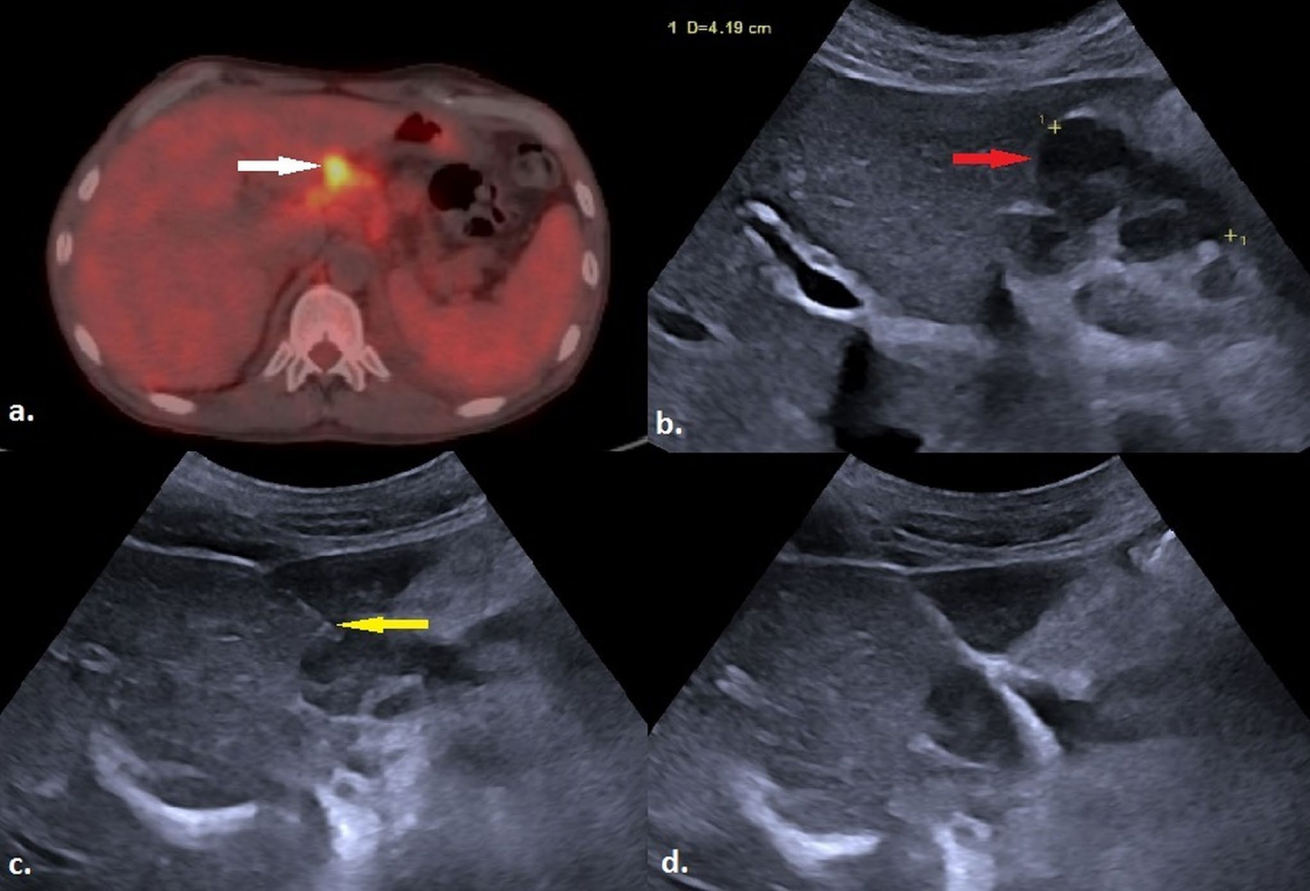
**a**) An axial PET/CT scan shows a nodular lesion (white arrow) with increased SUV uptake in the portal hilum. **b**) In the US image of the same lesion (red arrow), it appears as a lymphadenopathy with an identifiable echogenic hilum but displaying asymmetric cortical thickening and increased size. **c**) The lesion is accessed via CTHa using a 17-gauge introducer (yellow arrow). **d**) A US image obtained immediately after the biopsy procedure. Histopathological examination confirmed the lesion as Hodgkin lymphoma (PET/CT: Positron Emission Tomography / Computed Tomography; CTHa: Complete Transhepatic Approach; SUV: Standardized Uptake Value; US: Ultrasound)

To the best of our knowledge, no prior study has specifically examined the association between liver parenchyma length traversed in transhepatic biopsies and complication risk. Our findings indicate that while we observed only one major complication, a longer transhepatic pathway increased the likelihood of minor complications. Given that CTHa is already reserved for cases where no alternative route exists, it becomes even more critical to meticulously plan the biopsy trajectory to minimize the liver tissue traversed, as excessive liver parenchyma passage may unnecessarily elevate procedural risk. Furthermore, while multivariate analysis identified liver parenchyma length as an independent predictor of complications, univariate analysis also showed associations with malignancy and smaller lesion size along the biopsy trajectory. The association between a history of malignancy and increased complication risk is an expected finding, as previously reported in the literature [26, 27]. Unlike previous studies that have primarily focused on maximum lesion size or anterior-posterior dimensions [28, 29], our study is the first, to our knowledge, to introduce and emphasize the significance of lesion size specifically along the biopsy trajectory in the context of abdominal mass biopsies. This distinction is particularly relevant in anatomically challenging locations with restricted approach angles, as seen in transhepatic biopsies. Notably, while maximum lesion size had no impact on complication rates, a smaller lesion size along the biopsy trajectory significantly increased complication risk. By shifting the focus from overall lesion dimensions to the portion encountered along the biopsy path, our findings provide a novel perspective on procedural risk stratification and contribute to a more refined understanding of risk factors in abdominal core biopsies. Moreover, the term ‘transhepatic’ is often used inconsistently, sometimes referring to procedures that fully traverse the liver (e.g., percutaneous cholecystostomy) and at other times to those involving only a single capsular penetration (e.g., transhepatic liver parenchymal biopsy, percutaneous transhepatic cholangiography), which may create ambiguity. Given that our study specifically evaluates cases requiring full liver traversal, we believe that adopting the term ‘complete transhepatic approach (CTHa)’ provides a more precise and clinically relevant distinction (Fig. 8).

The pathology results of 8 out of 71 biopsies in our study were reported as nondiagnostic, corresponding to a nondiagnostic rate of 11.3%. Of these nondiagnostic biopsies, 6 were from lesions located in the porta hepatis and 2 were from the right para-aortic/paracaval regions. Additionally, among the 8 lesions reported as nondiagnostic, 7 were of mixed type containing cystic areas, while 1 was solid in nature. Although the difference was not statistically significant (*p* = 0.081), the average lesion size along the biopsy trajectory was smaller in the nondiagnostic group compared to the diagnostic group (22.0 ± 4.9 mm vs. 34.6 ± 18.2 mm). Given the anatomical characteristics of these locations, it is reasonable to assume that a considerable proportion of the lesions were lymph nodes. Several studies, including that by Picardi et al., have demonstrated that percutaneous core biopsy of lymph nodes achieves satisfactory diagnostic performance comparable to excisional/surgical biopsy, with reported nondiagnostic rates ranging from 3 to 13.7% [30–34]. However, a large-scale study by Syrykh et al. in 2022, which included 32,285 patients from the French Lymphopath network, found that lymph node core biopsies were associated with higher rates of erroneous and nondefinitive results compared to surgical biopsies and were more frequently referred for expert pathological review by referral centers [30]. We believe that the likely high proportion of lymph nodes among the lesions located in the porta hepatis and paracaval/paraaortic regions, combined with smaller lesion size, contributed to the relatively high nondiagnostic outcomes. Additionally, the presence of cystic components in a significant portion of these lesions may have further reduced the diagnostic yield. Furthermore, it is important to note that 750 of the biopsies in Picardi et al.’s study were obtained from superficial lymph nodes, and a similar distribution is likely in other studies focusing on lymph node biopsies. In contrast, the majority of the lymph node biopsies in our study involved deeply located intra-abdominal and retroperitoneal lesions, often in close proximity to major vascular structures such as the aorta, inferior vena cava (IVC), and main portal vein. Additionally, the requirement for a transhepatic approach and the need to maintain a limited biopsy angle due to these anatomical constraints likely increased the technical complexity of the procedure and contributed to our relatively higher nondiagnostic rate.

This study has several limitations. First, as a single-center retrospective study, it is inherently subject to the constraints of retrospective designs. Second, although the decision to utilize the transhepatic route was made following a thorough pre-procedural evaluation and consensus between two interventional radiologists, each with over five years of experience, the lack of standardized guidelines or an objective protocol defining the specific indications and contraindications for this approach remains a limitation. While CTHa is generally regarded by interventional radiologists as a technique reserved for challenging abdominal mass biopsies when no extrahepatic route is feasible, its broader adoption may be facilitated by larger, multicenter studies that establish clearer recommendations and guidelines. With better-defined contraindications and procedural frameworks, CTHa is likely to become a more frequently utilized technique, particularly for US-guided procedures. This issue is not unique to our study but rather a common challenge in research on alternative biopsy routes for complex abdominal lesions. Third, to assess long-term complications such as tract seeding, prolonged and regular follow-up of patients is necessary. In our study, some patients died during follow-up, while others discontinued follow-up at our institution, preventing a reliable evaluation of these long-term outcomes. Lastly, although three core samples were obtained from each lesion, the use of an 18-gauge needle may have contributed to our nondiagnostic rate. However, given that a coaxial system was employed, the use of a larger-caliber needle, such as a 16-gauge needle, would have necessitated a 15-gauge coaxial introducer, requiring two transgressions of the liver capsule, thereby potentially increasing the risk of complications. Therefore, the risk-benefit balance should be carefully considered when selecting the needle size in transhepatic biopsies.

## Conclusion

The CTHa under ultrasound guidance provides a technically feasible and low-risk option for biopsying deeply located abdominal masses when no extrahepatic route is available. When performed by experienced operators, it yields high diagnostic accuracy with a low complication rate. Our findings underscore the importance of minimizing liver tissue traversal to reduce minor complications and highlight the diagnostic challenges associated with lesions in anatomically complex regions such as the porta hepatis and paraaortic/paracaval areas. Given these considerations, the CTHa represents a valuable solution in anatomically demanding cases where conventional access routes are not feasible.

## Data Availability

No datasets were generated or analysed during the current study.
